# The neurotoxic threat of micro- and nanoplastics: evidence from In Vitro and In Vivo models

**DOI:** 10.1007/s00204-025-04091-3

**Published:** 2025-06-03

**Authors:** Ana Margarida Araújo, Carolina Mota, Helena Ramos, Miguel A. Faria, Márcia Carvalho, Isabel M. P. L. V. O. Ferreira

**Affiliations:** 1 LAQV/REQUIMTE, Bromatology and Hydrology Laboratory, Faculty of Pharmacy, University of Porto, Porto, Portugal; 2 FP-I3ID, FP-BHS, University Fernando Pessoa, Porto, Portugal; 3 RISE-Health, Faculty of Health Sciences, Fernando Pessoa University, Fernando Pessoa Teaching and Culture Foundation, Porto, Portugal

**Keywords:** Microparticles, Nanoparticles, Neuronal damage, Human health, Cell models, Animal models

## Abstract

Micro- and nanoplastics (MPs/NPs), ubiquitous contaminants in ecosystems and food chains, have emerged as a significant concern due to their potential neurotoxic effects on human health. Here, we conducted a systematic review of the existing literature, which included 26 studies providing evidence from cellular and animal studies on the risks posed by MPs/NPs to the nervous system. In vitro studies reveal that MPs/NPs can disrupt the integrity of the blood–brain barrier, penetrate neurons and glial cells, impair cell membrane integrity, and induce cytotoxic effects. These plastic particles trigger oxidative stress, inflammation, and mitochondrial dysfunction, alter signaling pathways, and disrupt neuronal communication, potentially leading to neurological dysfunction, cognitive deficits, and neurodegenerative disorders like Alzheimer’s and Parkinson’s diseases. Animal models corroborate these findings, demonstrating behavioural changes, memory impairment and neurotransmitter imbalances following exposure to MPs/NPs. Although the evidence in humans is limited, the growing body of hazard data underlines the potential risks associated with chronic exposure and accumulation of MPs/NPs in the nervous system. This highlights the urgent need for further research to elucidate the mechanisms of neurotoxicity, as well as stringent regulatory measures to restrain plastic pollution and safeguard neurological health.

**Supplementary Information:**

The online version contains supplementary material available at 10.1007/s00204-025-04091-3.

## Introduction

Plastics are leaving a lasting environmental footprint, infiltrating ecosystems worldwide and accumulating in diverse habitats. The very properties that make plastics so valuable across sectors – from agriculture and healthcare to construction, food packaging, and technology (Patil et al. 2022; Pironti et al. 2021) – such as their durability, resistance to weathering and mechanical damage (Bai et al. 2022; Kadac-Czapska et al. 2024), are the same characteristics that facilitate the persistence of plastic waste. Combined with suboptimal recycling practices (Jiang et al. 2020), plastics persists in the environment, and can break down into microscopic fragments, yielding microplastics (MPs) under 5 mm in size and nanoplastics (NPs) under 1 µm (Hartmann et al. 2019). These particles are small enough to be inadvertently ingested by a wide range of organisms, accumulating in tissues and even crossing biological barriers. From marine species to humans, the exposure risks associated with these particles are becoming increasingly concerning, highlighting the profound impact of plastics on health and ecosystems.

In addition to size, MPs/NPs can vary significantly in polymer type, and surface functionalization, and these characteristics may contribute to differences in their environmental and health impacts. These plastic particles are made from long-chain polymers such as polyethylene (PE), high-density polyethylene (HDPE), low-density polyethylene (LDPE), polyethylene terephthalate (PET), polyvinyl chloride (PVC), polystyrene (PS), and polypropylene (PP), and may contain additives such as plasticizers, stabilizers, and flame retardants (Agboola and Benson 2021). Different polymer types influence the persistence and ability of MPs/NPs to adsorb and transport various harmful environmental pollutants, such as persistent organic pollutants, heavy metals, and pharmaceuticals (Verla et al. 2019). Chemical interactions are driven by specific characteristics such as hydrophobicity and the presence of functional groups in their surface, which are formed during degradation. These groups, such as carboxyl (-COOH) and amine (-NH_2_), have the potential to increase toxicity and influence how particles interact with biological systems (Ma et al. 2024; Shi et al. 2022). For example, amino-modified NPs have been observed to adhere strongly to cells. This phenomenon may be due to electrostatic interactions that enhance cell uptake and internalization (Shi et al. 2022).

Human exposure to MPs and NPs can occur through inhalation, skin contact, eye contact and, most commonly, ingestion of contaminated food (Kadac-Czapska et al. 2024). Once in the body, they can enter the systemic circulation via the respiratory and digestive tracts, which may result in their accumulation in various tissues and organs (Bai et al. 2024a; Zhu et al. 2023). Given their minute size, NPs are uniquely suited to be internalized by blood–brain barrier (BBB) endothelial cells, potentially enabling them to translocate across this barrier. This raises significant concerns about the direct delivery of NPs and associated toxic substances to the brain.

The impact of plastic pollution on the central nervous system (CNS) has been the subject of considerable research in recent years, with studies conducted on a range of organisms, predominantly aquatic invertebrates (mostly clams, mussels and nematodes) and vertebrate (fish) species (see (Prust et al. 2020), for an extensive review). The results of these studies showed that exposure to MPs/NPs inhibited acetylcholinesterase (AChE) activity and increased oxidative stress, ultimately leading to significant damage to neuronal structure and function and behavioural changes. In contrast, there is a paucity of studies investigating the neurotoxicity of MPs/NPs in terrestrial mammals. The physiological characteristics and exposure routes of terrestrial vertebrate models differ from those of previous aquatic models. In aquatic species, the uptake of MPs/NPs occurs through the gills, as opposed to the oral ingestion and intestinal uptake or inhalation observed in mammals. Such differences influence the uptake and/or distribution of MPs/NPs and hinder the translation of effects observed in aquatic species to the characterization of neurotoxic hazard in mammals (Vorhees et al. 2021) and, ultimately, in humans. Consequently, rodents (mice and rats) represent a superior model for investigating the toxicological effects of MPs/NPs in humans (Vorhees et al. 2021). Additionally, various mammal and human cellular models play a crucial role in the comprehensive evaluation of the hazards posed by MPs/NPs.

The goal of this review was thus to perform a systematic search of literature focused on the current evidence on how MPs/NPs may cause damage to the human central nervous system (CNS) by scrutinizing studies using human and rodent cells as in vitro models and mammalian (rodent) in vivo models. This approach will enhance the cross-species translatability of findings to human biology. This study also aimed to identify and summarize the mechanisms of MPs/NPs-induced neurotoxicity and to highlight key gaps in our understanding that must be addressed to fully elucidate the risks they pose to humans.

## Methods

### Search strategy

This review was performed according to the Preferred Reporting Items for Systematic Reviews and Meta-Analysis (PRISMA) guidelines (PRISMA 2024). A rigorous search strategy was employed, including PubMed, Scopus, and the Web of Science databases. The search, conducted on June 11, 2024, included all studies published from database inception to June 6, 2024. The inclusion keywords for the review were as follows: “neurotoxic*” AND (“plastic nanoparticles” OR “plastic microparticles” OR “nanoplastic*” OR “microplastic*”) in the title or abstract. After optimization, additional exclusion keywords were added with the operator NOT: “review” (publication type), “aquatic”, “bivalve”, “mussel”, “earthworm”, “fish”, “Caenorhabditis”, “zebrafish”, and “plant*”. The detailed search strategy for each database, designed to maximize the retrieval of relevant articles, is presented in **Online Resource 1** in the Supplementary Material section.

### Inclusion and exclusion criteria

To ensure a clear and focused assessment of the direct effects of MPs/NPs on the nervous system, the criteria used to include and exclude studies were carefully defined. Inclusion criteria: Model systems: only studies using rodent and/or human models; Study design: both in vitro and in vivo studies were considered; Exposure: only studies specifically investigating the individual effects of MPs/NPs on the nervous system; Language: only papers written in English.

Exclusion criteria: studies involving co-exposure of MPs/NPs with other contaminants; review articles; studies using models other than mammals (rodents and humans); articles evaluating the toxicity of MPs/NPs in organ systems other than the nervous system; and papers focusing on the microbiota-gut-brain axis.

### Overview of included studies

**Fig. 1:**
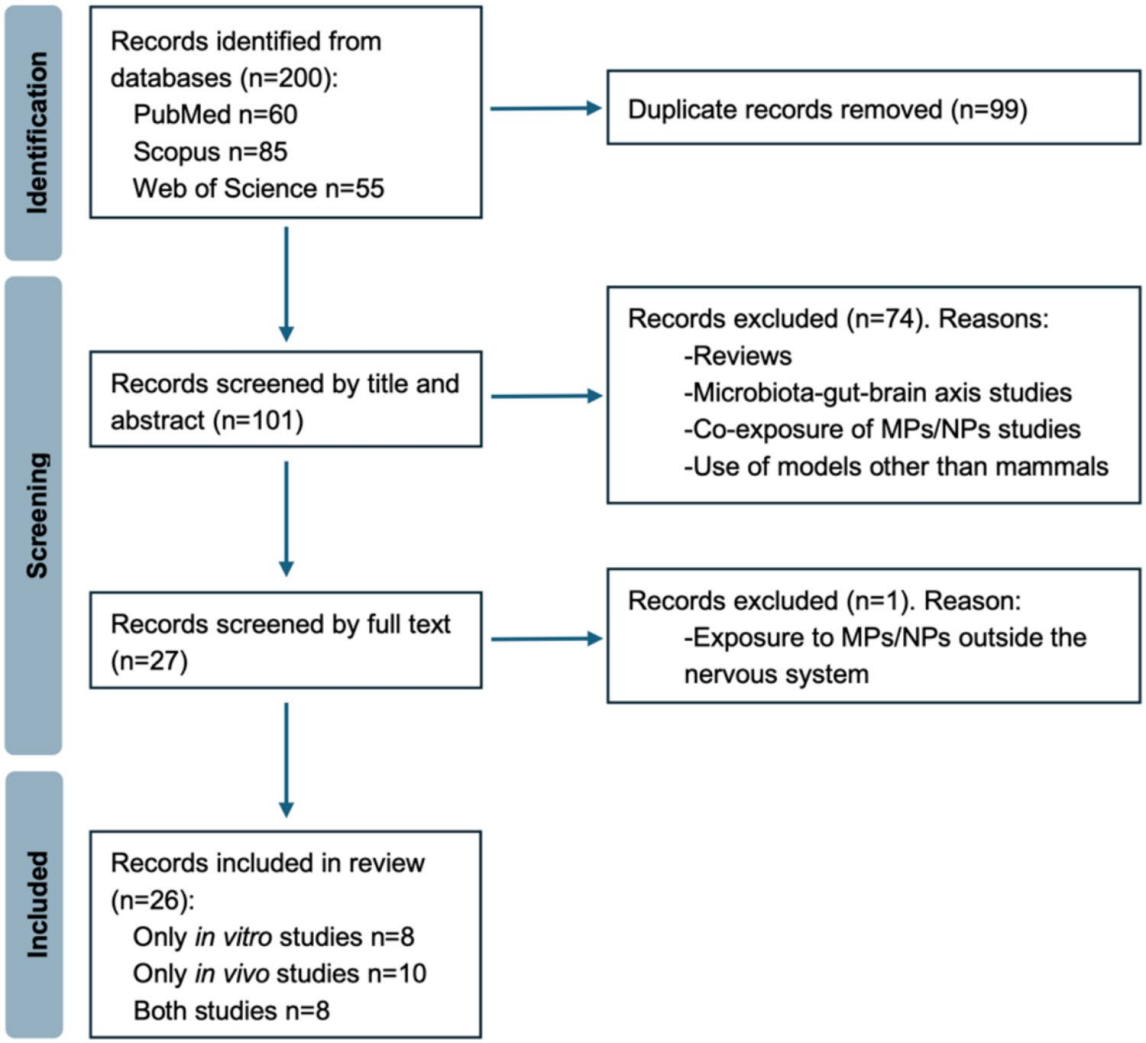
PRISMA flow diagram of study selection

The initial search yielded a substantial pool of 200 records. The EndNote 20 software was employed to eliminate duplicates, resulting in a refined set of 101 research articles for further review. An initial meticulous screening based on titles and abstracts resulted in the exclusion of 74 records. The full text of the remaining 27 papers was then reviewed, with 26 articles ultimately meeting the inclusion criteria considered for this systematic review. These articles encompassed both in vitro (16 papers) and in vivo (18 papers) studies. In some cases, a single article included both in vivo and in vitro findings, and these were considered in both experimental models. Figure 1 depicts the PRISMA flow diagram of the study selection process.

### Data extraction

Relevant information from the selected articles, including particle size and shape, functional groups, nervous system model, study conditions, key outcomes, and toxicity mechanisms, was collected and summarized in Tables 1 and 2.

**Table 1:** Summary of in vitro studies assessing the neurotoxic effects of micro- and nanoplastics included in this systematic review

Study	Particle size/Shape	Functional groups	Company	Nervous system model	Conditions	Outcomes	Toxicity mechanisms
Jung et al. 2020	100 nm NPs / Beads	None	Polyscience, Inc. (Warrington, PA, USA)	Mouse embryonic fibroblasts (MEFs), mixed neuronal cells isolated from embryonic cortex, and cortical astrocytes	100 and 200 µg/mL 2 days exposure	↓ Cell viability at 100 and 200 µg/mL in primary astrocytes ↑ Caspase-3 levels in a concentration-dependent manner in mixed neuronal cells ↑ *Lcn2* expression levels indicate reactive astrocytosis ↓ Tubb3 and ↑ Gfap expression levels in 100 µg/mL Intracellular accumulation of PS-NPs in a concentration-dependent manner ↑ Proinflammatory cytokines (TNF-α and IL-1β)	Cell apoptosis Inflammatory responses
Shan et al. 2022	50 nm NPs / NA	None	Bangs Laboratories, Inc. (Fishers, IN, USA)	Human cerebral microvascular endothelial cells (hCMEC/D3), murine microglia BV2 cells	25, 50 and 100 μg/mL 3 days exposure	hCMEC/D3 cells NF-kB activation**;** ↑ TNF-α levels; disruptions of tight junctions; ↓ TEER and expression of occludin; ROS generation in a concentration- and time-dependent manner BV2 cells PS-NPs accumulate in the cytoplasm; enlargement of the cell body; ↑ TNF-α and IL-1β levels; ↑ ROS generation	hCMEC/D3 cells Necroptosis BV2 cells Oxidative stress; microglial activation; neuronal damage
Hua et al. 2022	1 and 10 µm MPs / Spheres	None	Phosphorex, Inc. (Hopkinton, MA)	hiPSC-derived cortical spheroids	5, 50 and 100 μg/mL 30 days	From 4 to 10 days: ↑ expression of Ki67, MKI67, ATF4, Nestin, PAX6, HOXB4 and SOD2 From 4 to 30 days: ↓ Cell viability; ↓ Expression of TUBB3 and TBR1/TBR2	Cellular stress Cytotoxicity
Liu et al. 2022	100 nm NPs, 1 µm MPs / NA	None	Beijing DK nano technology *Co.LTD* (Beijing, China)	HT22 mouse hippocampal neuronal cells	5, 25 and 75 μg/mL 1 day exposure	↓ Cell viability in a concentration-dependent manner ↑ ROS generation S phase arrest particularly at 25 and 75 μg/mL	Oxidative stress Cell apoptosis
Tang et al. 2022	50 nm NPs / Beads	None	Janus New-Materials (Nanjing, China)	Human neuroblastoma cell line SH-SY5Y	20, 50, 100, 200 and 500 µg/mL 1 day exposure	↓ Cell viability, ↑ LDH and ROS release in a concentration-dependent manner Induction of mitochondrial damage ↓ ATP levels, particularly at 200, and 500 µg/mL ↑ Ca^2+^ and ↓ MMP at 100, 200, and 500 µg/mL ↑ Caspase-3, -9, Apaf-1 levels; ↑ of autophagy-related proteins LC3-Il, Beclin-1, Atg12, 15 and 16L ROS, LDH, MMP and apoptosis results reverted by NAC pre-treatment	ROS generation Oxidative stress Mitochondrial dysfunction Cell apoptosis Autophagy
Sun et al. 2023	44 nm NPs/Spheres	None	Bangs Laboratories, Inc	Mouse microglial cell line BV2	25, 50 and 100 μg/mL 12 h and 1 day exposure	↓ Cell viability ↑ ROS generation ↑ GPX4, XCT, ACSL4 ↑ NLRP3, and IL-1β in a concentration- and time-dependent manner ↑ ROS and MDA levels and ↓ GSH and SOD activities in a concentration- and time-dependent manner Modification of ferritin transport proteins ROS levels reverted by NAC pre-treatment	Oxidative stress Inflammatory responses Ferroptosis Lipid peroxidation
Yang et al. 2023	30 nm NPs, 50 nm NPs, 2 µm MPs / NA	PS-NH_2_, PS-COOH	Sigma-Aldrich (St. Louis, MO, USA) and Polysciences Inc. (Warrington, PA, USA)	C17.2 neuronal progenitor cells	100, 200 and 500 μg/mL 1- and 2-days exposure	↓ Proliferation in cells only treated with 50 nm PS at both time points All types of NPs with a size of 50 nm or smaller penetrated the cell membrane ↓ Expression of cyclin D and CDK4 PS-NH_2_ ↑ Expression of p-H2AX, p21, and p27; mitochondrial dysfunction; disruption of MMP; G1 phase cell cycle arrest; cellular senescence; DNA damage; 200 μg/mL of PS-NH_2_ caused ROS production ↑ Lipid and protein oxidation ↑ Expression of SASP-related genes (Il6, Ccl2, Ccl3, Mmp1 and Mmp3)	Cytotoxicity DNA damage Lipid and protein oxidation Mitochondrial dysfunction Cellular senescence Cell apoptosis
So et al. 2023	2 µm MPs / NA	None	SpheroTech (Shanghai, China)	Primary cortical neurons	1 and 10 µg/mL 3 days exposure	↓ length of primary dendrites Disruption of dendritic growth at 10 µg/mL, ↑ number of Ki-67 + and BrdU + cells ↑ cleaved caspase-3	Cell apoptosis
Huang et al. 2023	50 nm NPs / Spheres	None	Magsphere (Pasadena, CA, USA)	Human SH-SY5Y neuroblastoma cell line	0.5, 5, 50 and 500 μg/mL 2 days exposure	PS-NPs accumulated in the cytoplasm in a concentration-dependent manner ↓ Cell viability at 50 and 500 µg/mL ↑ ROS levels Morphological changes, mitophagosome formation, ↓ MMP and ATP levels; mitophagy via the AMPK/ULK1 pathway • NAC reduced ROS levels	Cytotoxicity ROS generation Oxidative stress Mitochondrial dysfunction
Wang et al. 2024	2.5 µm MPs / NA	None	NA	Mouse microglia BV2	1, 10 and 100 µg/mL 1 day exposure	↑ Production and release of TNF-α, IL-1β, and IL-6 in a concentration-dependent manner ↑ Pyroptosis related proteins N-GSDMD and GSDMD	Pyroptosis Inflammatory responses
Bai et al. 2024b	100 nm NPs / NA	PS-NH_2_	Tianjin Saierqun Technology Co. Ltd. (Tianjin, China)	hCMEC/D3 and mouse hippocampal neuronal cells (HT22)	CMEC/D3: 50 µg/mL HT22: 40 µg/mL 12 h exposure	hCMEC/D3 cells ↓ Cell viability; ↓ occludin and ZO-1 at 50 µg/mL; PS-NH_2_ ↑ TLR2 and MMP-9 levels HT22 cells ↓ Cell proliferation; ↑ Bax, iNOS and nNOS levels; ↓ Bcl-2 and NeuN levels; GAPDH/Ac-Tau signaling	hCMEC/D3 cells Inflammatory responses HT22 cells Cell apoptosis
Ma et al. 2024	100 nm NPs / NA	PS-COOH, PS-NH_2_	Tianjin Baseline Chromtech Research Center (Tianjin, China)	hCMEC/D3; human neuroblastoma cell line SH-SY5Y; mouse microglia cell line BV-2	30 µg/mL 1 day exposure	hCMEC/D3 cells PS-COOH and PS-NH_2_ ↓ ZO-1 and occludin levels; BBB impairment SH-SY5Y cells All types of NPS internalized in the mitochondria; suppression of mitochondrial respiration; ↓ ATP levels; ↑ *Drp1* gene expression particularly in the PS-COOH and PS-NH_2_ groups; ↓ anti-apoptotic Bcl-2 expression BV2 cells All types of NPs activated microglial; ↑ CD68-positive cells; ↓ CD206-positive cells; ↑ Inflammatory markers IL-1β, iNOS, and TNF-α	hCMEC/D3 cells BBB disruption SH-SY5Y cells ROS production; PS-COOH and PS-NH_2_ caused cell apoptosis; mitochondrial dysfunction; microglial activation BV2 cells Microglial activation; Inflammatory responses
Marcellus et al. 2024	50 and 500 nm NPs / Beads	None	Polysciences, Inc. (Warrington, PA, USA)	Rat fetal neural stem cells (rNSCs); rNSC-derived oligodendrocytes, astrocytes, and neurons	0.01, 0.1, 1, 10, 100 and 1000 μg/mL 1- and 7-days exposure	↓ Cell viability particularly with 500 nm NPs at 1000 μg/mL Impairment of lipid metabolism For 50 nm particles, many genes were upregulated, and more for 500 nm NPs	Cytotoxicity Cell apoptosis Inflammatory response
Li et al. 2024	480 ± 30.3 nm (hd) MPs / NA	None	Qiyue biological technology, China	Mouse microglia BV2	25, 50 and 100 μg/mL 1, 2, and, 3 days exposure	↓ Cell viability at 50 and 100 µg/mL ↑ NO and pro-inflammatory cytokines in concentration- and time-dependent manner ↑ HRAS, Iba-1, NF-kB p65, and p-PERK levels	Neuroinflammation Cytotoxicity
Paing et al. 2024	30 and 50 nm NPs/Spheres	None	NA	Mice primary cultures of microglia, astrocytes, and neurons	200 µg/mL 1 day exposure	Suppression of hippocampal neuronal activity Cognitive dysfunction Synaptic loss Brain pathologies ↑ CD86 activation LPS increases PS-NPs uptake Nuclear translocation of NF-kB	Microglial activation Inflammatory responses Neuroinflammation Cell apoptosis
Vojnits et al. 2024	20 and 100 nm NPs; 2 µm MPs/Fibers	None	Phosphorex (Hopkinton, MA, USA)	Human cortical and nociceptive neurons, derived from two types of human neural precursors (neural precursor cells from human bone marrow and neural precursor cells converted from human peripheral blood)	6 to 200 MPs/NPs per well 2 days exposure	↓ Cell viability, ↓ Neurite length and number in a concentration-dependent manner Strong axonal attachment, accumulation, and morphological chances even at low concentrations; ROS generation Human cortical neurons are more susceptible to toxicity of NPs than nociceptive neurons	Oxidative stress Cytotoxicity

All studies used polystyrene (PS) particles as the model plastic material

Abbreviations: *ACSL4* Achaete-Scute Family BHLH Transcription Factor 4, *Ac-Tau* Acetylated Tau, *AMPK/ULK1* adenosine monophosphate-activated protein kinase / UNC-51 like kinase 1, *Apaf-1* Apoptotic protease activating factor-1, *ATF4* Activating transcription factor 4, *ATP* Adenosine triphosphate, *BBB* Blood–Brain Barrier, *Caspase-3* Cysteinyl aspartate specific proteinase-3, *Caspase-9* Cysteinyl aspartate specific proteinase-9, *CD86* Cluster of differentiation 86, *CDK4* Cyclin-dependent kinase 4, *GAPDH* Glyceraldehyde 3-phosphate dehydrogenase, *Gfap* Glial fibrillary acidic protein, *GPX4* Glutathione peroxidase 4, *GSDMD* Gasdermin D, *GSH* Glutathione, *hCMEC/D3* human cerebral microvascular endothelial cell line, *hd* hydrodynamic diameter, *Iba-1 * Ionized calcium-binding adapter molecule 1, *IL-1β* Interleukin 1β, *IL-6* Interleukin 6, *iNOS* Inducible nitric oxide synthase, *hiPSC* human induced pluripotent stem cells K3, *Lcn2* Lipocalin-2, *LDH* Lactate dehydrogenase, *LPS* Lipopolysaccharide, *MDA* Malondialdehyde, *MEFs* Mouse embryonic fibroblasts, *MMP* Mitochondrial membrane potential, *MMP-9* Matrix metalloproteinase-9, *NA* Not Available, *NAC* N-acetylcysteine, *NF-kB* Nuclear factor kappa B, *NLRP3* NOD- LRR- and pyrin domain-containing protein 3, *NO* Nitric oxide, *NPs* Nanoplastics, *p-PERK* phosphorylated-protein kinase RNA-like endoplasmic reticulum kinase, *PS-COOH* carboxyl-modified PS-NPs, *PS-NH*_*2*_ amino-modified PS-NPs, *PS-NPs* Polystyrene nanoplastics, *ROS* Reactive oxygen species, *SASP* senescence-associated secretory phenotype, *SOD* Superoxide dismutase, *TBR1* T-box brain transcription factor 1, *TEER* Transendothelial electrical resistance, *TLR2* Toll-like receptor 2, *TNF-α* Tumour necrosis factor α, *Tubb3* Tubulin β-III, *XCT* Solute carrier family 7 member 11, *ZO-1* Zonula Occludens-1, ↑ Increased, ↓ Decreased

**Table 2:** Summary of in vivo studies assessing the neurotoxic effects of micro- and nanoplastics included in this systematic review

Study	Particle size/Shape	Functional groups	Company	Nervous system model	Conditions	Outcomes	Toxicity mechanisms
Rafiee et al. 2018	25 and 50 nm NPs / NA	None	Kisker (Germany)	Male Wistar rats	1, 3, 6 and 10 mg/kg/day 35 days exposure	Anxiety behaviour ↓ Locomotor activity at 3 mg/kg/day dose, ↑ Avoidance latency	Cognitive deficits
Liang et al. 2022	50 nm NPs/Spheres	None	Magsphere (Pasadena, CA, USA)	Male C57BL/6 J mice	0.25, 2.5, 25 and 250 mg/kg 28 days exposure	Downregulated pathways associated with neurodegenerative diseases Mitochondrial and synaptic dysfunction ATP metabolism impairment	Cell apoptosis Inflammatory responses
Wang et al. 2022	5 µm MPs/NA	None	Shanghai Macleans Biochemical Co., Ltd	Male Kunming mice (KM)	0.01, 0.1 and 1 mg/day 30 days exposure	↑ Escape latency in a dose-dependent manner ↓ Ratio of brain weight and body weight Cellular disorganization Pyramidal cells damage and ↓ Nissl bodies ↓ GSH and ↑ MDA levels; ROS generation ↑ Total SOD activity in higher doses ↓ Acetylcholine levels; ↑ AchE and ChAT activity Vitamin E treatment reduced the severity of adverse effects	Oxidative stress Cognitive deficits
Shan et al. 2022	50 nm NPs/NA	None	Bangs Laboratories, Inc. (Fishers, IN, USA)	Male C57BL/6 J mice	0.5, 2.5, 10 and 50 mg/kg 7 days	↓ PECAM-1 expression ↑ Iba-1 expression ↑ BBB permeability	Microglial activation Neuronal damage
Chu et al. 2022	25 nm NPs/NA	None	Bangs Laboratories, Inc. (Fishers, IN, USA)	Male C57BL/6 mice	10, 25 and 50 mg/kg 6 months exposure	↑ Memory errors and incorrect movements Impairment in spatial learning and memory Vascular blockage, neuronal vacuolization, and cellular injuries, particularly at 50 mg/kg Deregulation of 96 mRNAs Synaptic damage ↑ ROS generation	Oxidative stress DNA damage Neuroinflammation Cognitive dysfunction
Han et al. 2023	100 nm NPs/NA	None	Polysciences, Warrington, PA, USA	Male CD-1 (ICR) mice	200 µg/mL 3 h and 1 day	At 3 h, ↑ levels of Gfap and ↓ levels of the neuronal marker Tubb3 ↑ expression levels of Lcn2 that cause neuronal cell death ↑ IL1-β and TNF-α only after 3 h HDAC6 inhibition relieves PS-NP-induced neurotoxicity and ↓ levels of the oxidative stress marker Hmox1	Cell apoptosis
Kang et al. 2023	80 nm NPs/NA	None	Baseline Chromatography Technology Development Center, Tianjin, China	C57BL/6 J mice	10 mg/mL 42 days	Altered expression of neurotransmitter levels (5-HT, GABA) and nervous system proteins (AChE, BDNF, SYN, CREB) ↓ learning and memory ability Changes in circadian pathways ↑ Camk2g and Adcyap1 mRNA, ↓ Per1 mRNA in mouse hippocampus Melatonin reduced nerve damage	Cells apoptosis Hippocampal damage and neurological dysfunction
Bas et al. 2023	350–500 nm MPs / NA	None	NA	Wistar albino rats	25 and 50 mg/kg 30 days exposure	↑ POD and GST levels Disruption of brain sexual dimorphism Behavioural issues	Oxidative stress
Yang et al. 2023	50 nm NPs and 2 µm MPs / NA	PS-NH_2_	Sigma-Aldrich (St. Louis, MO, USA)	Male C57BL/6 mice	50 and 200 mg/kg 10 days exposure	At 200 mg/kg, ↓ number of BrdU-positive cells and expression levels of nestin ↓ Cell proliferation Mitochondrial dysfunction Memory impairment	Cell apoptosis
Sharma et al. 2023	50 nm NPs / NA	None	Kisker, Germany (cat#PPS-0.050)	Male Swiss albino mice	0.2 and 1 mg/kg/day 56 days exposure	↓ locomotor activity Anxiety behaviors and learning impairments Cognitive function and memory impairment ↓ Antioxidant enzyme activities (SOD and CAT); GSH, VAChT, GAD, and SYP levels in a dose-dependent manner ↓ AchE activity	Oxidative stress Lipid peroxidation
So et al. 2023	2 µm MPs/NA	None	SpheroTech (Shanghai, China)	Male C57BL/6 mice	1 µg/mL 168 days exposure	Impaired social behaviour in the offspring No differences in expression levels of genes related to brain cell markers or synaptic organization	Not specified
Wang et al. 2024	2.5 µm MPs/Spheres	None	NA	C57BL/6 J mice and APP/PS1 double-transgenic Alzheimer’s disease mice	1, 10, or 100 mg/kg 28 days exposure	Cognitive deficits ↑ Activation and pyroptosis of central microglial cells ↑ IL-1β, IL-6, and TNF-α levels in a dose-dependent manner ↑ Pyroptosis executor protein GSDMD	Neuroinflammation Pyroptosis
Chen et al. 2024	50 nm NPs/NA	None	Beijing Zhongkeleiming Technology Co., Ltd	Pregnant Sprague–Dawley (SD) rats	2.5 mg/kg/day 43 days exposure	Upregulation of PZP, α-2-M and FN1 Alterations on neuron growth and development in the hippocampal CA3 region; ↑ inhibitory proteins PZP, α-2 M, FN1 and SERPINA1 Impairment of synaptic and neuronal functions ↓ DMTN expression ↑ Alox15 and transferrin proteins expression	Neuronal damage Cell apoptosis Neuronal ferroptosis
Bai et al. 2024b	100 nm NPs/Spheres	PS-NH_2_	Tianjin Saierqun Technology Co. Ltd. (Tianjin, China)	Male C57BL/6 mice	40 mg/kg/day 105 days exposure	↑ Escape latency ↓ Spatial learning and cognitive flexibility Memory and cognitive impairments Upregulation of AD mRNA markers Neuronal shrinkage and necrosis ↑ Bax levels; ↓ Bcl-2 and NeuN levels ↑ iNOS, nNOS, Ac-Tau expression; ↓ Sirt1 expression Adverse effects alleviated by the administration of Camellia pollen	Cell apoptosis
Suman et al. 2024	500 nm MPs / NA	None	Sigma Aldrich (L 3280)	Male Swiss albino mice	0.1, 1 and 10 ppm 28 days exposure	↓ Body weight at 1 ppm-PS; ↓ number of normal cells and Nissl bodies ↓ Neuron and spine density at 1 ppm at 1 and 1 ppm doses, disruption of dendritic arborization, ↓ number of Nissl bodies ↓ BDNF expression Impairment of synaptic activity and morphology at 1 ppm dose	Oxidative stress Cognitive and neurological impairments Neurophysiological changes
Ma et al. 2024	100 nm NPs/NA	PS-COOH, PS-NH_2_	Tianjin Baseline Chromtech Research Center (Tianjin, China)	Male BALB/c mice	10 mg/mL 3 days exposure	Anxiety and depression behaviours All types of NPs, but particularly PS-NH_2_ affected locomotor activity All types of NPs, but particularly PS-NH_2_ and PS-COOH altered social behaviour PS-COOH and PS-NH_2_ had more severe effects on neuronal damage BBB disruption Mitochondrial energy metabolism dysfunction; ↓ ATP production	Cell apoptosis Neuronal damage
Li et al. 2024	480 ± 30.3 nm (hd) MPs/Spheres	None	Qiyue biological technology, China	Male C57BL/6 J mice	2 and 10 mg/kg 7 days exposure	Anxiety behaviour in a dose-dependent manner ↓ Neurons and enlargement of cellular gaps ↓ Dendritic spine density in a dose-dependent manner ↑ TNF-α, IL-1β, IL-6 levels ↑ HRAS, Iba-1, NF-kB p65 and p-PERK levels	Inflammatory response Microglial activation Cell apoptosis
Paing et al. 2024	30 and 50 nm NPs/Spheres	None	NA	Male C57BL/6 J mice	10 and 20 mg/kg/day 49 days exposure	↓ Latency times ↑ Expression of CD86 Impairment of short-term and long-term memory Inhibition of neuronal activity in the hippocampus Learning and memory deficits	Oxidative stress Cognitive dysfunction Microglia activation

All studies used polystyrene (PS) particles as the model plastic material

Abbreviations: *5-HT* Serotonin, *α-2-M* alpha-2-macroglobulin, *AchE* Acetylcholinesterase, *Adcyap1* Adenylate cyclase-activating polypeptide 1, *ATP* Adenosine triphosphate, *BBB* Blood–brain barrier, *BDNF* Brain-derived neutrophic factor, *BrdU* 5-bromo-2’-deoxyuridine, *Caspase-3* Cysteinyl aspartate specific proteinase-3, *CAT* Catalase, *ChAT* Choline acetyltransferase, *CREB* cAMP-response element binding protein, *DMTN* Dematin actin-binding protein, *DNA* Deoxyribonucleic acid, *FN1* fibronectin 1, *GABA* γ-aminobutyric acid, *GAD* Glutamic acid decarboxylase, *Gfap* Glial fibrillary acidic protein, *GSDMD* Gasdermin D, *GSH* Glutathione, *GST* Glutathione S-transferase, *hd* hydrodynamic diameter, *HDAC6* Histone deacetylase 6, *Hmox1* Heme oxigenase 1, *Iba-1* Ionized calcium-binding adapter molecule 1, *IL-1β* Interleukin 1 beta, *IL-6* Interleukin 6, *iNOS* Inducible nitric oxide synthase, *KM* Kunming, *Lcn2* Lipocalin-2, *MDA* Malondialdehyde, *NA* Not available, *NF-kB* Nuclear factor kappa B, *PECAM-1* Platelet endothelial cell adhesion molecule-1, *Per1* Period circadian regulator 1, *POD* Peroxidase enzyme, *p-PERK* Phosphorylated-protein kinase RNA-like endoplasmic reticulum kinase, *PS-COOH* carboxyl-modified PS-NPs, *PS-NH*_*2*_ amino-modified PS-NPs, *PS-NPs* Polystyrene nanoplastics, *PZP* Pregnancy-zone protein, *ROS* Reactive oxygen species, *Sirt1* Sirtuin 1, *SOD* Superoxide dismutase, *SYN* Synaptophysin, *TNF-α* Tumour necrosis factor alfa, *Tubb3* III β-tubulin, *VAChT* Vesicular acetylcholine transporter, ↑ Increased, ↓ Decreased

## Results and discussion

### Evidence of neurotoxic effects of MPs/NPs—Insights from in vitro studies

In vitro studies on the neurotoxicity of MPs/NPs have focused exclusively on polystyrene (PS) polymers, using a range of primary and immortalized neuronal cell models. The most used were the mouse BV2 microglial cell line (5 studies), stem cells (4 studies), the human SH-SY5Y neuroblastoma cell line (3 studies), the human cerebral microvascular endothelial cell line (hCMEC/D3) (3 studies), mouse primary cell cultures (3 studies), and the mouse hippocampal neuronal cell line (HT22) (2 studies). Each model has distinct advantages and limitations. Endothelial cells serve as a BBB model but lack the neuronal-glial complexity. Mouse embryonic fibroblasts mixed with neurons and glial cells provide physiological relevance but lack human specificity. Human neuroblastoma cells provide a human-based model, but do not fully mimic normal neuronal function. Stem cells, although costly and complex, provide more human-relevant toxicological insights, enhancing research accuracy (Fritsche et al. 2021). The studies identified in the literature search are summarized in Table 1 and discussed below.

### Human cerebral microvascular endothelial cell line (hCMEC/D3)

Studies in the human cerebral microvascular endothelial cell line (hCMEC/D3) highlight the impact of polystyrene nanoplastics (PS-NPs) on the integrity of the BBB, although they investigate different aspects of NP properties and their effects. Shan et al. (2022) demonstrated that PS-NPs (50 nm) induce ROS production, activate NF-κB and promote necroptosis, leading to BBB disruption via tight junction alterations (Shan et al. 2022). Bai et al. (2024b) focused on amino-modified PS-NPs (PS-NH_2_, 100 nm) and showed reduced cell viability and a pronounced inflammatory response characterized by elevated TLR2 and MMP-9 as well as downregulation of tight junction proteins (Bai et al. 2024b). Ma et al. (2024) investigated the effects of different types of PS-NPs (PS, PS-COOH, PS-NH_2_, 100 nm) and showed that while cell viability was largely unaffected, there were alterations in tight junctions and BBB permeability, with functionalized PS-NPs having more significant effects (Ma et al. 2024). Taken together, these works highlight the importance of NP size, surface modification and exposure time in BBB disruption, with different mechanisms of inflammation and tight junction protein modulation.

### Murine Microglial BV2 cell line

The BV2 microglial cell line is the most commonly used model for studying PS-MPs/NPs neurotoxicity, with consistent findings across studies indicating pro-inflammatory responses. Shan et al. (2022) linked PS-NP accumulation to ROS production, cytokine release, and neuronal damage via microglia-derived factors (Shan et al. 2022). Sun et al. (2023) further implicated ferroptosis, showing oxidative stress and lipid peroxidation as key mechanisms, while Li et al. (2024) identified the PERK-NF-κB pathway in inflammation, with HRAS silencing mitigating effects (Li et al. 2024). Wang et al. (2024) uniquely demonstrated pyroptosis induction, highlighting Gasdermin D activation (Wang et al. 2024). Expanding beyond plain PS particles, Ma et al. (2024) showed that surface-modified PS-NPs influenced microglial polarization toward a pro-inflammatory M1 phenotype (Ma et al. 2024). Collectively, these studies reinforce PS-MPs/NPs-induced neuroinflammation, emphasizing their potential neurotoxic risk.

### Human neuroblastoma SH-SY5Y cell line

Research using SH-SY5Y cells consistently demonstrate PS-NPs-induced neurotoxicity, with oxidative stress and mitochondrial dysfunction as key mechanisms. Tang et al. (2022) linked ROS production to apoptosis and autophagy, highlighting NAC’s protective role. Extending exposure to 2 days, Huang et al. (2023) confirmed similar oxidative damage but uniquely identified AMPK/ULK1-mediated mitophagy and Complex I dysfunction, leading to dopaminergic neuron death. Ma et al. (2024) introduced surface-modified NPs, showing that while all variants disrupted mitochondrial function, PS-COOH and PS-NH_2_ induced stronger Drp1-mediated fission and apoptosis. Collectively, these studies highlight oxidative stress, apoptosis, and mitochondrial impairment as key neurotoxic pathways of PS-NPs.

### HT22 mouse hippocampal neuronal cell line

Liu et al. (2022) demonstrated that PS-NPs (100 nm) induce sustained ROS production, cell cycle arrest, and reduced viability in HT22 cells, whereas larger PS-MPs (1 μm) had minimal effects (Liu et al. 2022). Bai et al. (2024b) further highlighted the impact of surface modifications, showing that PS-NH_2_ (100 nm) triggered apoptosis via Bax/Bcl-2 imbalance and nitric oxide synthase signaling (Bai et al. 2024b). These studies emphasize the role of size in PS-induced neurotoxicity and reveal distinct toxicity mechanisms between plain and functionalized PS-NPs.

### Mice primary cells cultures

Studies on mouse brain primary cells reveal distinct cell type-specific responses to PS-MPs/NPs. Jung et al. (2020) showed that while mouse embryonic fibroblasts (MEFs) were resistant, mixed neuronal cells from the embryonic cortex were highly susceptible to apoptosis, and astrocytes exhibited a reactive phenotype with increased proinflammatory markers (Jung et al. 2020). In contrast, So et al. (2023) found that larger PS-MPs (2 µm) did not enter neurons but impaired early dendritic growth and induced both proliferation and apoptosis (So et al. 2023). Paing et al. (2024) highlighted microglia as primary responders to smaller PS-NPs (30–50 nm), showing activation, increased uptake under inflammatory conditions, and suppression of neuronal activity (Paing et al. 2024). Together, these studies underscore the diverse neurotoxic effects of PS particles, with neurons, astrocytes, and microglia exhibiting distinct but interrelated responses, warranting further investigation into long-term neurotoxic risks.

### Stem cells

Stem cell models provide human-relevant insights into PS-MPs/NPs neurotoxicity, revealing cell type-specific vulnerabilities. Marcellus et al. (2024) demonstrated that astrocytes were the most sensitive among rat neural stem cells (rNSCs) and differentiated cells, with 500 nm PS inducing stronger gene expression changes despite lower cytotoxicity (Marcellus et al. 2024). Vojnits et al. (2024) showed that human cortical neurons were highly susceptible to MPs, while nociceptive neurons displayed resistance, with low-density cultures more affected by NPs (Vojnits et al. 2024). Both studies emphasize the heightened toxicity of smaller particles.

Expanding on surface modifications, Yang et al. (2023) found that only cationic PS-NPs (50 nm) induced toxicity in mouse neural progenitor cells (NPCs), causing ROS-driven DNA damage, mitochondrial dysfunction, and G1 cell cycle arrest—effects not seen with neutral or anionic PS-NPs. This highlights the role of charge and size in toxicity mechanisms (Yang et al. 2023).

Moving to a 3D model, Hua et al. (2022) used human cortical spheroids, showing that prolonged exposure to 1 µm PS-MPs disrupted neurodevelopment by impairing cell adhesion and downregulating neuronal markers (Hua et al. 2022). Importantly, this study underscores the necessity of considering 3D models for neurotoxicity assessments.

### Evidence of neurotoxic effects of MPs/NPs—Insights from in vivo studies

This section summarizes key findings from in vivo studies of the neurotoxic effects of PS-MPs and PS-NPs in rodents, with an emphasis on potential mechanisms. The literature review shows a predominant use of the C57BL/6 mouse model (n = 9), with Wistar rats and Swiss albino mice used in two studies each. Other models, including Kunming (KM) mice, CD-1 (ICR) mice, APP/PS1 double transgenic Alzheimer’s disease (AD) mice, Sprague–Dawley (SD) rats and BALB/c mice, were reported in one study each. The detailed results are presented in Table 2 and the key findings are summarized below.

### Wistar rats

The first in vivo study investigating the neurotoxic effects of PS-NPs (25 and 50 nm) found dose-dependent behavioral changes in Wistar rats, including altered anxiety levels and reduced locomotion, but no significant memory impairment (Rafiee et al. 2018). Expanding on this, Bas et al. (2023) used higher doses of PS-NPs (350–500 nm) and identified hippocampal neuronal degeneration, with notable sex-specific effects. Female rats exhibited altered grooming behavior and oxidative stress marker variations, while males remained largely unaffected (Bas et al. 2023). These results suggests estrogen signaling and brain sexual dimorphism may influence NP-induced neurotoxicity, highlighting the need for sex-based analyses in future research.

### Sprague–Dawley (SD) rats

While most studies focus on the immediate neurotoxicity of PS-NPs in adult rodents, Chen et al. (2024) uniquely investigated transgenerational effects. Maternal PS-NPs exposure led to proteomic alterations in offspring, including upregulated inhibitory proteins (PZP, α-2 M, FN1, SERPINA1) linked to neural damage and downregulated transport-related proteins (KIF21A, STMN2), potentially impairing synaptic function. Notably, decreased expression of dematin actin-binding protein (DMTN) suggests an increased risk of brain tumors, while elevated Alox15 and transferrin levels indicate ferroptosis involvement. These findings highlight the long-term neurodevelopmental risks of prenatal PS-NPs exposure.

### C57BL/6 mice

PS-NPs exposure in C57BL/6 mice consistently demonstrate BBB permeability, neuroinflammation, and cognitive impairment, with variations in exposure duration, dose, and particle size revealing distinct mechanisms.

Shan et al. (2022) first established that PS-NPs (50 nm, 7 days) crossed the BBB, accumulating in microglia and causing neuronal damage (Shan et al. 2022). Extending exposure to 28 days, Liang et al. (2022) linked PS-NPs to Parkinson’s disease-related pathways, showing mitochondrial dysfunction and synaptic disruption, with pronounced effects in the substantia nigra and striatum (Liang et al. 2022). Paing et al. (2024) further highlighted microglia activation as a driver of cognitive deficits but found no motor impairments (Paing et al. 2024), unlike Liang et al. (2022). Longer exposure (6 months) by Chu et al. (2022) confirmed persistent learning and memory deficits, correlating with oxidative stress, neuroinflammation, and a complex ceRNA network affecting synaptic function (Chu et al. 2022).

Beyond cognition, Kang et al. (2023) identified hippocampal damage and neurotransmitter imbalances in C57BL/6 mice exposed to PS-NPs (80 nm), emphasizing circadian rhythm disruption as a novel mechanism (Kang et al. 2023). Larger PS-MPs (2 µm) studied by So et al. (2023) did not impair memory but altered social behaviors, suggesting subtler neurodevelopmental effects (So et al. 2023). Li et al. (2024) revealed PS-MPs-induced anxiety via the HRAS-PERK-NF-κB pathway, marking a distinct inflammatory mechanism (Li et al. 2024).

Surface modifications further shaped toxicity. Short-term exposure to cationic PS-NH_2_ impaired hippocampal neurogenesis (Yang et al. 2023), while long-term exposure induced AD-like neurodegeneration, characterized by Tau accumulation and apoptotic signaling (Bai et al. 2024b).

Collectively, these studies highlight oxidative stress, neuroinflammation, and mitochondrial dysfunction as central to PS-NP toxicity, with exposure duration, particle size, and surface chemistry influencing specific neurotoxic outcomes.

### Swiss albino mice

Studies on Swiss albino mice consistently show neurotoxic effects of subchronic PS-NPs exposure, though with variations in dosage, duration, and particle size. Sharma et al. (2023) demonstrated that 50 nm PS-NPs (0.2–1 mg/kg/day, 8 weeks) impaired locomotion, increased anxiety, and disrupted cholinergic signaling, with oxidative stress and neuronal injury across multiple brain regions (Sharma et al. 2023). In contrast, Suman et al. (2024) examined 500 nm PS-NPs (0.1–10 ppm, 28 days) and found a non-linear accumulation pattern in the brain, reduced neuronal density, dendritic atrophy, and BDNF downregulation, particularly at 1 ppm (Suman et al. 2024). While both studies confirm PS-NPs-induced neurotoxicity, Sharma et al. (2023) highlight neurotransmitter-related damage, whereas Suman et al. (2024) emphasize morphological and dendritic alterations, suggesting distinct yet overlapping neurotoxic pathways.

### Kunming mice

Wang et al. (2022) used the Kunming mouse model to investigate the impact of oral PS-MPs (5 μm, 0.01–1 mg/day) on learning and memory. They observed a dose-dependent cognitive decline, accompanied by hippocampal structural damage, including irregular cell arrangement, pyramidal cell injury, and dendritic alterations. Biochemically, PS-MPs induced oxidative stress, reducing GSH levels, increasing MDA and ROS, and activating total superoxide dismutase (T-SOD). Additionally, PS-MPs disrupted cholinergic function, lowering acetylcholine levels and altering acetylcholinesterase and choline acetyltransferase activity. Notably, PS-MPs also reduced phosphorylated cAMP-response element binding protein (CREB) levels, a key protein in cognitive processes, highlighting their potential neurotoxic impact (Wang et al. 2022).

### APP/PS1 double-transgenic Alzheimer’s disease mice

Wang et al. (2024) investigated the impact of PS-MPs (2.5 µm, 28 days) on cognitive function in APP/PS1 double-transgenic Alzheimer’s mice, finding that higher doses of PS-MPs exacerbated cognitive deficits, as indicated by increased escape latency and reduced movement (Wang et al. 2024). This was accompanied by increased neuroinflammation, with elevated levels of cytokines (IL-1β, IL-6, TNF-α) and increased microglial pyroptosis, as evidenced by higher expression of gasdermin D (GSDMD). Notably, inhibiting GSDMD alleviated PS-MPs’ effects, highlighting pyroptosis as a key mechanism. This study provides novel insights into PS-MPs as potential environmental risk factors for AD progression (Wang et al. 2024).

### CD-1 (ICR) mice

Han et al. (2023) examined the effects of intranasally administered 100 nm fluorescent PS-NPs in male ICR mice, highlighting significant changes within 3 and 24 h. In contrast to previous studies, which mainly focused on long-term effects, Han et al. observed a rapid increase in the astrocyte marker Gfap and a decrease in the neuronal marker Tubb3 after just 3 h, indicating early neuroinflammation. Furthermore, the study reported a rise in the neurotoxin Lcn2 and inflammatory markers IL-1β and TNF-α, pointing to acute neurotoxic responses. Notably, the study introduced HDAC6 inhibition as a novel approach to reduce oxidative stress and neurotoxicity by enhancing PS-NP clearance. These findings underscore the fast action of PS-NPs in the brain and their potential neuroinflammatory risks (Han et al. 2023).

### BALB/c mice

Ma et al. (2024) investigated the effects of PS-NPs, PS-COOH, and PS-NH_2_ (100 nm, 3 days) on male BALB/c mice, revealing that all NPs crossed the BBB and accumulated in the brain. While no significant changes in body weight or brain index were observed, exposure led to anxiety-like and depression-like behaviors, particularly with PS-NH_2_. Notably, altered social behavior and reduced sociability were linked to these neurotoxic effects. The study further identified mitochondrial accumulation of NPs, disrupting energy metabolism and decreasing ATP production, offering novel insight into the link between NP exposure and behavioral changes (Ma et al. 2024).

## Conclusions and future research priorities

The evidence gathered from in vitro studies has consistently demonstrated that PS-MPs/NPs induce cytotoxicity, oxidative stress, mitochondrial dysfunction, cellular integrity disruption, and neuroinflammation. Common effects include microglial activation, increased ROS production, inflammatory responses, and particle accumulation within neural cells. These particles also compromise the BBB, exacerbating neural damage. Functionalized NPs exhibit heightened toxicity, including DNA damage and cell cycle arrest. Smaller particles and higher concentrations lead to more severe cytotoxic effects, raising concerns about the greater health risks posed by NPs compared to MPs.

**Fig. 2:**
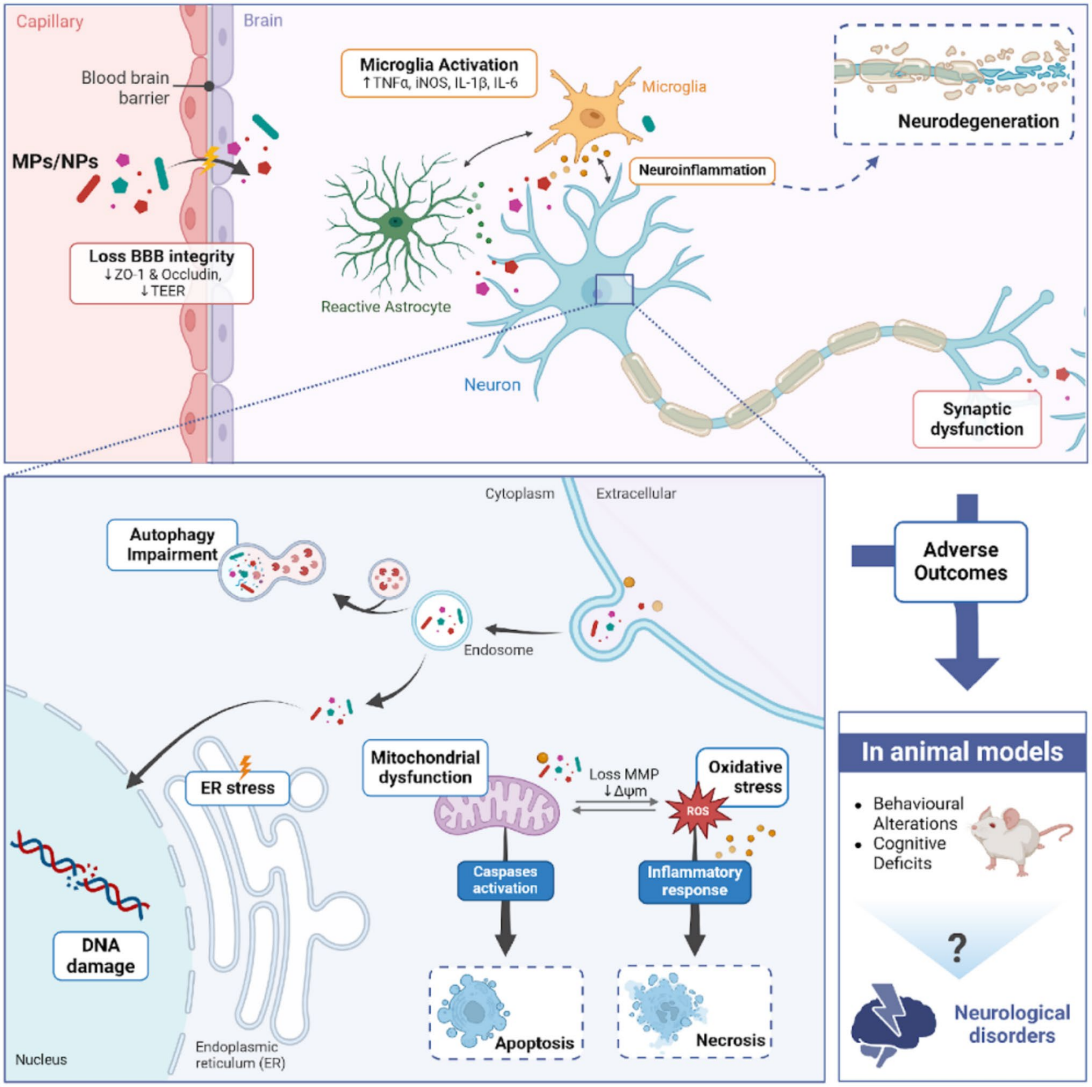
Mechanisms of micro- and nanoplastics (MPs/NPs)-induced neurotoxicity. The scheme illustrates the multifactorial processes through which MPs/NPs disrupt brain function and lead to neurotoxicity. MPs/NPs penetrate the blood–brain barrier (BBB), resulting in a loss of BBB integrity marked by reduced levels of tight junction proteins like ZO-1 and occludin, alongside increased transendothelial electrical resistance (TER). After entrance, MPs/NPs activate microglia and astrocytes, promoting neuroinflammation through upregulation of inflammatory cytokines (TNFα, IL-1β, IL-6, and iNOS). Neuroinflammation subsequently aggravates neurodegeneration processes. Within neuronal cells, MPs/NPs impair intracellular pathways, including autophagy. Further downstream effects involve DNA damage, endoplasmic reticulum (ER) stress, mitochondrial dysfunction (loss of mitochondrial membrane potential), and oxidative stress driven by reactive oxygen species (ROS) and reduction of antioxidant defenses. These processes trigger caspase activation, inflammatory responses, and ultimately result in cellular apoptosis and necrosis. The adverse outcomes demonstrated in animal models, include behavioral alterations and cognitive deficits. Therefore, the potential neurotoxicity of MPs/NPs and their role in the development of neurological disorders in humans warrant consideration

In vivo studies corroborate these findings, showing consistent adverse effects across different particle sizes, types, exposure durations, and administration routes. All studies revealed a dose-dependent relationship, with higher doses or prolonged exposures causing more severe outcomes. Even low doses of MPs/NPs resulted in behavioral and cognitive impairments. Smaller particle size emerged as a key factor in toxicity. Figure 2 summarizes the main mechanisms of MPs/NPs-induced neurotoxicity reviewed in this work.

This review highlights the urgent need for stricter regulations to reduce plastic pollution and safeguard neurological health, emphasizing the importance of coordinated efforts in scientific research and public policy. However, significant research gaps remain. The following topics outline key areas for improving the accuracy of MPs/NPs risk assessments.

- **Chronic low-dose exposure:** much of the current research has focused on acute exposure models. Future studies should investigate chronic low-dose exposures, which may better reflect real-world conditions due to the ubiquitous presence of plastic particles in the environment and consumer products. This will help to elucidate the cumulative effects on neurons, synaptic connections, and cognitive function over time, enhancing long-term neurotoxicity risk assessments in humans.
- **Diverse polymer types**: current studies mainly focus on PS-MPs/NPs. Future research should investigate the toxicity of PS-MPs/NPs in combination with other environmental polymers, such as PET, PP, PVC, and PE, which differ in environmental durability and capacity to adsorb and transport toxic substances. Investigating these combinations with other environmental contaminants (e.g., heavy metals, persistent organic pollutants, and pesticides) is critical to understanding real-world exposure scenarios and their synergistic or antagonistic neurotoxic effects.
- **Functionalized PS-MPs/NPs:** there is limited research on functionalized PS-MPs/NPs. Given their modified surfaces, these particles may be more efficiently taken up by cells and distributed throughout the body, potentially leading to more severe consequences. Future studies should focus on how particle size, shape, surface charge, and polymer type influence toxicity and bioaccumulation in neuronal cells, providing insights that could inform regulatory standards for particle size and concentration.
- **Naturally aged particles:** most toxicity studies have used pristine MPs/NPs, but environmental MPs/NPs often undergo weathering and biofilm formation. This may increase toxicity through altered surface characteristics or the release of toxic additives and pollutants. Comparative studies on synthetic versus naturally aged MPs/NPs are needed to assess how environmental aging affects toxicity profiles in neuronal cells.
- **Molecular mechanisms and therapeutic interventions:** although MPs/NPs are known to disrupt the BBB and induce oxidative stress, inflammation, and apoptosis, more research is needed to elucidate the specific molecular cascades involved. Future studies should also explore therapeutic and nutritional interventions aimed at mitigating the adverse effects of exposure or targeting neuroinflammation.
- **Advanced models for neuronal interactions:** to better model the complexity of neuronal networks, future research should include advanced in vivo models and 3D neural cultures. Animal studies can provide insights into how MPs/NPs affect brain function and behavior, while 3D neuronal cultures and organoids offer a more human-relevant model for studying plastic toxicity.

## Supplementary Information

Below is the link to the electronic supplementary material.

Supplementary file1 (DOCX 17 KB)

## Data Availability

Data sharing not applicable to this article as no datasets were generated during the current study.
