# Supplementary material for: The neurotoxic threat of micro- and nanoplastics: evidence from In Vitro and In Vivo models
Source: Arch Toxicol. 2025 Jun 3;99(9):3505–25. doi: 10.1007/s00204-025-04091-3 (PMC12408696; doi:10.1007/s00204-025-04091-3)
Supplement: Supplementary file 1 — Supplementary file1 (DOCX 17 KB) [file 204_2025_4091_MOESM1_ESM.docx]

# **Supplementary Material – Archives of Toxicology**

# **The Neurotoxic Threat of Micro- and Nanoplastics: Evidence from *In Vitro* and *In Vivo* Models**

Ana Margarida Araújo^1*#^, Carolina Mota^1#^, Helena Ramos^1^, Miguel Faria^1^, Márcia Carvalho^1,2,3*^, Isabel M.P.L.V.O. Ferreira^1^

^1^LAQV/REQUIMTE, Bromatology and Hydrology Laboratory, Faculty of Pharmacy of the University of Porto, Portugal

^2^ FP-I3ID, FP-BHS, University Fernando Pessoa, Porto, Portugal

^3^ RISE-Health, Faculty of Health Sciences, Fernando Pessoa University, Fernando Pessoa Teaching and Culture Foundation, Porto, Portugal

*Corresponding authors: Ana Margarida Araújo ([amaraujo@ff.up.pt](mailto:amaraujo@ff.up.pt)) and Márcia Carvalho ([mcarv@ufp.edu.pt](mailto:mcarv@ufp.edu.pt))

# both authors contributed equally to the manuscript

**Online Resource 1.** Search strategies used in this study.

| Database | Search strategy | N results |
| --- | --- | --- |
| PubMed | ((((((((("neurotoxic*"[Title/Abstract] AND ("plastic nanoparticles"[Title/Abstract] OR "plastic microparticles"[Title/Abstract] OR "nanoplastic*"[Title/Abstract] OR "microplastics"[MeSH Terms])) NOT "review"[Publication Type]) NOT "aquatic"[Title/Abstract]) NOT "bivalve*"[Title/Abstract]) NOT "mussel*"[Title/Abstract]) NOT "earthworm*"[Title/Abstract]) NOT "fish"[Title/Abstract]) NOT "Caenorhabditis"[Title/Abstract]) NOT "zebrafish"[Title/Abstract]) AND 2019/06/01:2024/06/06[Date - Publication] AND "english"[Language] | 60 |
| Scopus | (TITLE-ABS-KEY ( neurotoxic* ) AND TITLE-ABS-KEY ( "plastic nanoparticles" OR "plastic microparticles" OR nanoplastic* OR microplastic* ) ) AND NOT ( TITLE-ABS-KEY ( "plant s" OR planted OR planting OR plantings OR plant OR plants ) ) AND NOT ( TITLE-ABS-KEY ( aquatic OR mussel* OR bivalve* OR earthworm* OR fish OR caenorhabditis OR zebrafish) ) AND ( PUBYEAR > 2019 AND PUBYEAR < 2025 ) AND ( LIMIT-TO ( DOCTYPE , "ar" ) ) AND (LIMIT-TO(LANGUAGE, "English") | 85 |
| Web of Science | TS=(neurotoxic*)  AND TS=("plastic nanoparticles" OR "plastic microparticles" OR nanoplastic* OR microplastics)  NOT TS=("plant s" OR planted OR planting OR plantings OR plants OR plant)  NOT TS=(aquatic OR mussel* OR bivalve* OR earthworm* OR fish OR Caenorhabditis OR zebrafish)  AND PY=(2019-2024)  AND DT=(Article)  AND LA=(English) | 55 |
